# Enhancement of hybridoma formation, clonability and cell proliferation in a nanoparticle-doped aqueous environment

**DOI:** 10.1186/1472-6750-8-3

**Published:** 2008-01-14

**Authors:** Natalie Gavrilov-Yusim, Ekaterina Hahiashvili, Marina Tashker, Victoria Yavelsky, Ohad Karnieli, Leslie Lobel

**Affiliations:** 1 Department of Virology and Developmental Genetics, Ben Gurion University of the Negev, Beersheva 84105, Israel; 2 Department of Human Molecular Genetics and Biochemistry, Sackler School of Medicine, Tel Aviv University, Tel Aviv 69978, Israel

## Abstract

**Background:**

The isolation and production of human monoclonal antibodies is becoming an increasingly important pursuit as biopharmaceutical companies migrate their drug pipelines away from small organic molecules. As such, optimization of monoclonal antibody technologies is important, as this is becoming the new rate-limiting step for discovery and development of new pharmaceuticals. The major limitations of this system are the efficiency of isolating hybridoma clones, the process of stabilizing these clones and optimization of hybridoma cell secretion, especially for large-scale production.

Many previous studies have demonstrated how perturbations in the aqueous environment can impact upon cell biology. In particular, radio frequency (RF) irradiation of solutions can have dramatic effects on behavior of solutions, cells and in particular membrane proteins, although this effect decays following removal of the RF. Recently, it was shown that nanoparticle doping of RF irradiated water (NPD water) produced a stabilized aqueous medium that maintained the characteristic properties of RF irradiated water for extended periods of time. Therefore, the ordering effect in water of the RF irradiation can now be studied in systems that required prolonged periods for analysis, such as eukaryotic cell culture. Since the formation of hybridoma cells involves the formation of a new membrane, a process that is affected by the surrounding aqueous environment, we tested these nanoparticle doped aqueous media formulations on hybridoma cell production.

**Results:**

In this study, we tested the entire process of isolation and production of human monoclonal antibodies in NPD water as a means for further enhancing human monoclonal antibody isolation and production. Our results indicate an overall enhancement of hybridoma yield, viability, clonability and secretion. Furthermore, we have demonstrated that immortal cells proliferate faster whereas primary human fibroblasts proliferate slower in NPD water.

**Conclusion:**

Overall, these studies indicate that NPD water can enhance cell proliferation, clonability and secretion. Furthermore, the results support the hypothesis that NPD water is effectively composed of stable microenvironments.

## Background

Emerging therapeutic strategies are becoming increasingly dependent on immunotherapeutic approaches. In particular, this consists of humanized or human monoclonal antibody production, which is largely dependent on cell-based technologies. Development of human monoclonal antibodies is achieved with a couple of different techniques, one of which involves the formation of human hybridoma cells. Our laboratory isolates human monoclonal antibodies from peripheral blood lymphocytes with the hybridoma technique, using both humanized and fully human fusion partner cell lines [1,2]. Hybridoma cells, like primary cell cultures, are exquisitely sensitive to their environment [3-5] and require conditioned media that contains various stimulatory and growth factors for stabilization [6]. Indeed, like non-transformed cells, they clone very poorly, and combined with poor stability, this has become the limiting factor in the ability to continually produce any human monoclonal antibody (and murine for that matter too), which is identified in a primary hybridoma culture.

The formation of hybridoma cells involves the fusion of two cells and therefore the production of a new lipid bilayer membrane surrounding the contents of two cells. Similar to the formation of artificial lipid vesicles in an aqueous buffer system *in vitro *[7], the aqueous environment likely has an impact on these similar yet distinct processes. Biological systems, in general, are dependent on the aqueous environment, as life itself has evolved as a function of the properties of water. Biological processes from division of living cells to enzymatic reactions and DNA replication are intimately associated with, and dependent upon, the properties of water [8]. However, the aqueous foundation of life is, for the most part, taken for granted and most assume that it is a uniform medium [9]. As such, questions concerning the role that water plays in the *in vitro *processes of experimental biology have to a large extent been avoided in most biological studies. Thus, much of experimental biology rests upon the assumption that the aqueous environment of life is not an important factor in the outcome of most experimentation. This has resulted, thus far, in little investigation into the effect of the physical properties of water on biological systems.

Previous studies of water have demonstrated that irradiation in the microwave range of frequencies changes certain physical properties of water, likely generating water of a higher ordered structure [10,11]. However, this change is ephemeral and lasts only a couple of hours following removal of the irradiation. Recently, it was discovered that "nanoparticle-doping" of this irradiated (RF) water (NPD water) could stabilize the altered environment for extended periods of time [12]. As such, it is now possible to examine the effect of this novel aqueous environment on systems that require prolonged periods, such as days, for analysis. This new aqueous "formulation" has several unique properties including a probable higher ordered structure [12]. In many ways it is akin to doping of silicon in the semiconductor industry for production of silicon wafers with unique conductivities [13].

Recent studies in electrochemistry [12] have revealed some interesting properties of NPD water. These experiments suggest that our conventional view of water may have overlooked hidden properties that become apparent under unique conditions. Specifically, patterns of electrochemical deposition are altered [12], providing a dramatic demonstration of the effect of environmental order on chemistry. In the field of biology, studies have demonstrated significant changes in the opening frequency of potassium channels in cells grown in RF treated medium [14-16] and that bacteria have an increased growth rate when grown in RF treated medium [17].

Since isolation of human monoclonal antibodies is becoming pivotal for developing new immunotherapies, evaluation of technologies for enhancing the process of the chemical fusion technique [18] and optimization of its efficiency to yield a larger number of hybridoma cells has been an ongoing pursuit. These studies examine whether alterations in the aqueous environment impact on hybridoma cell formation and antibody secretion. They demonstrate that NPD water enhances the entire process of human monoclonal antibody isolation and production. Furthermore, proliferation studies on immortal and primary cells support the hypothesis that NPD water is effectively composed of stabilized microenvironments.

## Methods

### Materials

#### RF treated nanoparticle doped water

The effects of RF-treatment of water can be amplified and stabilized by doping with small quantities of crystalline nanoparticles (nanoparticle doped water (NPD)). This NPD water was prepared according to patent #PCT/IL2005/000198 (FDA DMF file number 20503), and as described by Katsir et. al. [12], and kindly provided by Docoop Technologies (Or Yehuda, Israel). The process utilizes ultrapure DI water that is kept below the "density anomaly point" (i.e. just below 4°C) and is radiofrequency irradiated (RF) at 915 MHz with a power of 60 watts. After 10 minutes of RF irradiation, sub-micron sized powder of barium titanate, which is heated to 900°C, is dropped from a furnace into the water and the RF-irradiation is continued for an additional five minutes. The treated water is then maintained at room temperature for two days, at which point it has clarified as most of the source powder (that contains larger particles) is separated at the bottom by gravity. The clarified treated water is then filtered through 0.22 micrometer filters to remove source particles of large size. Whereas the source powder for this process contains large agglomerates composed of small particles with rectangular and faceted shapes, after the doping process most of the particles that remain suspended in the water have almost perfect spherical shape. These observations indicate that during the production some of the large agglomerates disintegrate and that some of the individual particles alter their shape and become spherical. This effect is reminiscent of the phenomena observed during sonochemical synthesis of nanoparticles using cavitation [12,19,20].

The nanoparticle doped water that was produced by the above process for these experiments, was doped with spherical nanoparticles (approximately 10–50 nm size) of barium titanate at 10^15 ^particles per liter, which stabilizes the effect of RF on water [12]. As a control we used 18.2 mega ohm ultrapure deionized water (DI water, UHQ PS, ELGA Labwater) that is the starting material for preparation of the nanoparticle stabilized RF treated water. As a further control, we also tested DI water that was doped with nanoparticles without irradiation. All water was filtered through a 0.22 μm filter prior to use. Experiments with DI water doped with nanoparticles without irradiation yielded similar results to DI water in all experiments (data not shown) and we therefore present only experimental results of DI water versus NPD water in these studies for simplicity.

#### Reagents for cell growth

All the media and supplements for cell growth were purchased from GIBCO BRL, Life Technologies. RPMI 1640 and DMEM were purchased in powder form and reconstituted either in NPD or in DI water. After reconstitution sodium bicarbonate was added to the media according to the manufacturers' recommendation, and there was no further adjustment of pH. Prior to use, all the media were filter-sterilized through a 0.22 μm filter (Millipore). For the growth of hybridoma cells, RPMI was supplemented with 10% fetal calf serum, L-glutamine (4 mM), penicillin (100 U/mL), streptomycin (0.1 mg/mL), MEM-vitamins (0.1 mM), non-essential amino acids (0.1 mM) and sodium pyruvate (1 mM). All the supplements mentioned above were bought in a liquid form and used as is from the manufacturer (meaning, they were diluted into the NPD or DI based media). 8-Azaguanine, HT and HAT were purchased from Sigma and reconstituted from powder form with NPD or DI RPMI. DMEM used for human primary fibroblasts and CHO cells growth was supplemented with 10% fetal calf serum, L-glutamine (4 mM), penicillin (100 U/mL), streptomycin (0.1 mg/mL). Hybridoma cloning factor was bought from BioVeris.

#### Chemical reagents

Powdered PBS was obtained from GIBCO BRL, Life Technologies. PEG-1450 (P5402, Sigma) was purchased from Sigma and reconstituted with sterile PBS based on NPD or on DI water (50% w/v). The preparation was adjusted to pH 7.2, DMSO (v/v)(Sigma) was added to 10% followed by sterile filtration of the PEG solution through a 0.45 μm filter (Millipore). Hanks balanced salt solution was bought from Biological Industries Beit-HaEmek LTD, Israel and used as is for NPD and DI based experiments. Carbonate-bicarbonate buffer (0.05 M, pH = 9.6) for ELISA plate-coating, OPD (used in 0.4 mg/mL) and phosphate-citrate buffer (0.05 M, pH = 5.0) were bought from Sigma.

#### Antibodies

Goat anti-human IgM/IgG and HRP-conjugated goat anti-human IgM/IgG were purchased from Jackson ImmunoResearch. Standard human IgM/IgG were bought from Sigma.

#### Cells

All cells used in these experiments (MFP-2, CHO and primary human fibroblasts) were maintained for a week in either NPD or DI based media so that the cells were adapted to the media prior to experimentation. In addition, the fusion partner cell line MFP-2 [1] was maintained in RPMI 1640 with the addition of fetal bovine serum and additives as previously described [1] along with 8-azaguanine to maintain the HGPRT minus phenotype. Primary human fibroblasts were obtained from the ATCC and maintained in DMEM. The CHO cell line was maintained in DMEM. All cell culture was performed in complete media, which consists of culture media with the addition of fetal calf serum, glutamine and penicillin/streptomycin. For the MFP-2 cell line vitamins, nonessential amino acids and pyruvate were also added in complete medium.

### Methods

#### Cell Fusion

We employ the chemical fusion technique [18] with PEG 1450, which acts as a fusogen, for creation of hybridoma cells with human peripheral blood lymphocytes. PEG 1450 is typically prepared in PBS with the addition of 10% DMSO. For these experiments, NPD water was used to prepare PBS, which was then used to make a PEG/DMSO solution; as a control preparation we used PEG prepared in DI based PBS. For all fusion experiments comparing NPD to DI water, all reagents were prepared in either NPD or DI water except for fetal bovine serum and concentrates of supplements. In addition, dilution of cells in Hanks balanced salts (HBSS)(see below), following fusion with PEG-1450, was performed with a purchased liquid form of HBSS (Beit HaEmek, Israel) and used as is from the manufacturer. We preferred not to prepare this mixture ourselves as a minor deviation from its salt composition can introduce error when comparing fusion in NPD and DI water.

For production of hybridoma cells, human peripheral blood mononuclear cells (PBMC) were isolated from 40 mL of freshly drawn whole blood, purified with Histopaque 1077 (Sigma) as previously described, and washed 4 times in DI based culture medium without serum. The MFP-2 fusion partner cells were either grown in NPD or DI based media and then washed with the respective media 4 times without serum. For each experiment a single batch of PBMC was divided into two equal fractions, one of which was used for NPD and the other for DI fusions. Next, MFP-2 and PBMC were mixed in either NPD or DI based media without serum and pelleted. PEG-1450 pre-warmed to 37°C was then added at 300 μL for 10–200 × 10^6 ^of mixed cells. The cell mixture was incubated with PEG for 3 minutes with constant shaking. PEG was then diluted out of the cell mixture with Hanks balanced salt solution and complete RPMI (prepared in either NPD or DI water). To the resultant cell suspension were added: fetal calf serum (10%) and HT (×2). The hybridoma cells that were generated in this process were cultured in 96-well plates (cell density – 2 × 10^6 ^lymphocytes/well) in complete RPMI with HAT selection. The screening of the supernatants for immunoglobulin production was performed after the hybridoma cells occupied approximately 1/4 of the well.

#### Sandwich ELISA

A sandwich ELISA was used to screen hybridoma supernatants for IgM/IgG. Briefly, a capturing antibody (goat anti-human IgM/IgG) was prepared in a carbonate/bicarbonate buffer and applied on a 96-well plate in a concentration of 100 ng/100 μL/well. The plate was then incubated overnight at 4°C. All the following steps were performed at room temperature. After 1 hour of blocking with 0.3% dry milk in PBS, the supernatants from the hybridoma cells were applied for 1.5 hours. Human serum diluted 1:500 in PBS was used as a positive control. For a background and as a negative control hybridoma growth medium was used. The secondary antibody (HRP-conjugated goat anti-human IgM/IgG) was prepared in blocking solution at a concentration of 1:5000 and incubated for 1 hour. To produce a colorimetric reaction the plates were incubated with OPD in phosphate-citrate buffer, containing 0.03% H_2_O_2_. The color reaction was stopped with 10% HCl after 15 minutes. The reading and the recording of the reaction were performed with a Multiscan-Ascent (Thermo Scientific) ELISA reader using the 492 nm wavelength filter. All reagents used were standard with the exception of the sandwich layer, which consisted of the NPD or DI based hybridoma supernatant.

#### Cloning

Two hundred cells of a chosen clone were diluted in a volume of 10 mL of media and seeded in a 96-well plate (100 μL/well), so that on average the wells contained 1–2 cells. The cells were incubated and periodically fed and microscopically monitored for clonal growth. When a clone occupied 1/4–1/2 of the well, its supernatant was analyzed. The efficiency of cloning was expressed in a number of viable clones per plate. Ten percent HCF (hybridoma cloning factor) was added according to the experimental design.

#### Cell growth assay

Growth of primary and immortalized cell lines was monitored with a crystal violet dye retention assay as previously described [21]. A fixed number of cells was seeded in 96-well plates in multiple repeats. Cell growth was stopped by fixation in 4% formaldehyde. Fixed cells were then stained with 0.5% crystal violet followed by extensive washing with water. The retained dye was extracted in 100 μL/well of 0.1 M sodium citrate in 50% ethanol (v/v). The absorbance of the wells was then read at 550 nm with a Multiscan-Ascent microplate reader and the appropriate filter.

#### Primary human fibroblast culture

Starting at passage twenty, human fibroblasts were cultured and passed every week as long as the cells displayed typical fibroblast morphology and their number did not drop below the initially seeded amount. The number of passages and calculated population doublings were recorded. The morphology and viability of the cells were monitored microscopically. Human fibroblasts used in these experiments were generally at a population doubling of 25.

#### Data analysis

The statistical significance of difference in the efficiency of fusion and cloning between NPD and DI based experiments was determined by the Chi-square test. The results of the growth test with primary human fibroblasts were analyzed by an unpaired Students' t-test. Statistical p-values < 0.05 were considered significant.

## Results

### Nanoparticle doped RF treated water (NPD water) enhances efficiency of hybridoma formation for production of human monoclonal antibodies

**Figure 1:**
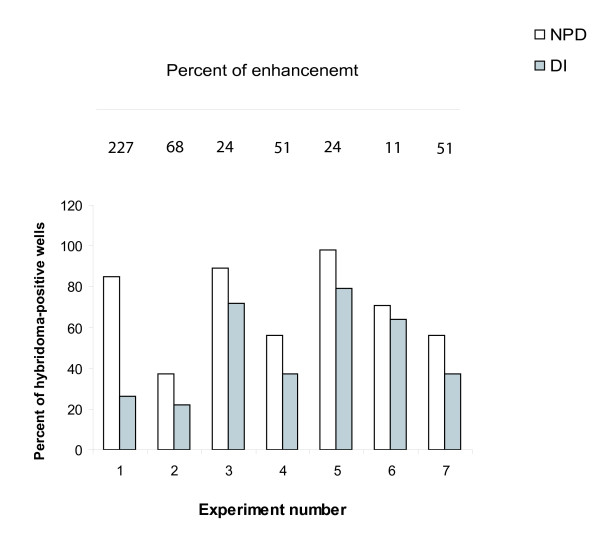
**Fusion efficiency enhancement**. The fusions were performed according to a standard protocol, where the culture media and PEG were reconstituted from powder forms with either NPD or DI water. For each fusion, PBMC from a single batch were divided into two equal fractures and used to prepare two parallel experiments, in NPD or DI based reagents. The figure presents percent of hybridoma-positive wells in each fusion experiment. The percent was calculated as the number of hybridoma-positive wells from 96-well plates where the cells were seeded and grown after the fusion process. The difference between the NPD- and DI-fusion results was found to be statistically significant by Chi-square analysis (p << 0.05) for all experiments. The percent of enhancement was calculated by the formula [(number of hybridomas in NPD-fusion/number of hybridomas in DI-fusion) x100-100%].

Results of chemical fusion experiments are presented in Figure 1. For these experiments PBMCs from a single individual were divided into two groups after purification for fusion in either a NPD or DI based environment. In our experiments we witnessed a statistically significant difference in the yield of hybridoma cells between NPD and DI environments. There was a clear tendency for a greater yield of hybridoma cells in the NPD based fusion experiments as compared to the parallel fusions in DI based media. The percent of enhancement was calculated by the formula [(number of hybridoma cells in NPD fusion/number of hybridoma cells in DI fusion) x100%-100%] and these results are depicted in Figure 1. The extent of enhancement is variable, and within a series of eight fusion experiments varied from 22 to 227 percent. Although the increased efficiency of fusion in NPD is variable, this is not unexpected as each fusion was performed with lymphocytes from a different donor. As such, magnitude of the effect of a NPD aqueous environment on hybridoma formation is a function to some extent of the genetic background.

### Increased yield of hybridoma subclones in NPD water

One of the crucial steps in the process of monoclonal antibody production is the isolation of a stable subclone from a primary hybridoma population found to be positive for secretion of a specific monoclonal antibody. This is typically achieved by serially subcloning of a specific primary hybridoma clone. The purpose of subcloning, which involves seeding 1–2 cells per well, is to produce clones of a single origin, which are genetically stable and produce a unique monoclonal antibody. During this process, hybridoma cells can die due to genetic instability or proliferate but lose their capacity to produce antibodies. To overcome these difficulties we use hybridoma cloning factor (HCF), which consists of macrophage conditioned media [6] containing a variety of factors that facilitate clone outgrowth and stabilization. However, since the fusion partner cell line we use is of myeloma origin [1], the hybridoma cells that are produced with it likely secrete autocrine factors that promote their own clonal expansion [22-24]. The autocrine action of these factors, however, is not apparent in standard *in vitro *culture due to their relatively low concentration. We therefore tested the hypothesis that NPD based media enhances the bioavailability, and hence autocrine activity, of these secreted factors, through increase in the cell-localized concentration. This was best achieved through subcloning primary hybridoma cells in DI versus NPD based media and also observing the effect of adding HCF to both cloning medias.

**Figure 2:**
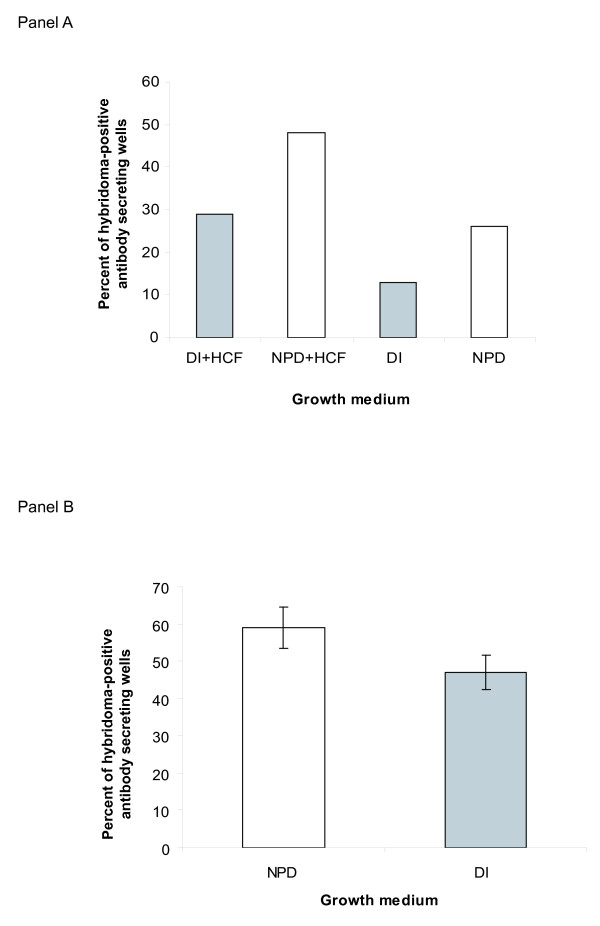
Panel A: Cloning efficiency of a primary hybridoma clone: From a single primary antibody-producing hybridoma clone 200 cells were plated per 96 well dish in replicates (on average 1–2 cells/well). The bar graphs present percentage of viable antibody producing subclones (from all plated wells) for each treatment. All differences were statistically significant by Chi-square analysis at p << 0.05 except for DI-RPMI+HCF versus NPD-RPMI, where the difference was not significant. Panel B: Cloning efficiency of a semi-stable hybridoma clone: From an antibody-producing semi-stable clone, 200 cells were counted and seeded over a 96-well plate (on average 1–2 cells/well). As this was a single semi-stable clone, unlike a primary hybridoma population, the experiment was performed multiple times and the data combined. The figure presents a mean percent of antibody-producing positive wells from replicate cloning experiments. The error bars denote the standard error of the mean.

Following fusion of PBMC with MFP-2 and outgrowth of primary hybridoma clones, antibody-producing hybridoma populations were identified and subcloned in either NPD or DI based media with supplements. The results of these experiments are displayed in Figure 2. Overall, for primary hybridoma populations tested, we observed greater clonal outgrowth of antibody secreting hybridoma cells in NPD based media as compared to DI based media. When HCF was added to both NPD and DI based media we saw a similar percentage increase in the number of antibody producing clones in both formulations. Figure 2 panel A illustrates a representative cloning experiment for a primary hybridoma population from five independent cloning experiments. All displayed the same trend where the number of clones in DI based media with HCF was statistically the same as that observed in NPD based media without added HCF. As primary hybridoma populations are highly unstable and each is different, data cannot be combined from multiple experiments nor multiple primary hybridoma population sublconings. Finally, as shown in Figure 2 panel B, clonability of cells from a semi-stable clone is also enhanced in NPD based media.

### Increased secretion of monoclonal antibodies from hybridoma cells grown in NPD water

**Figure 3:**
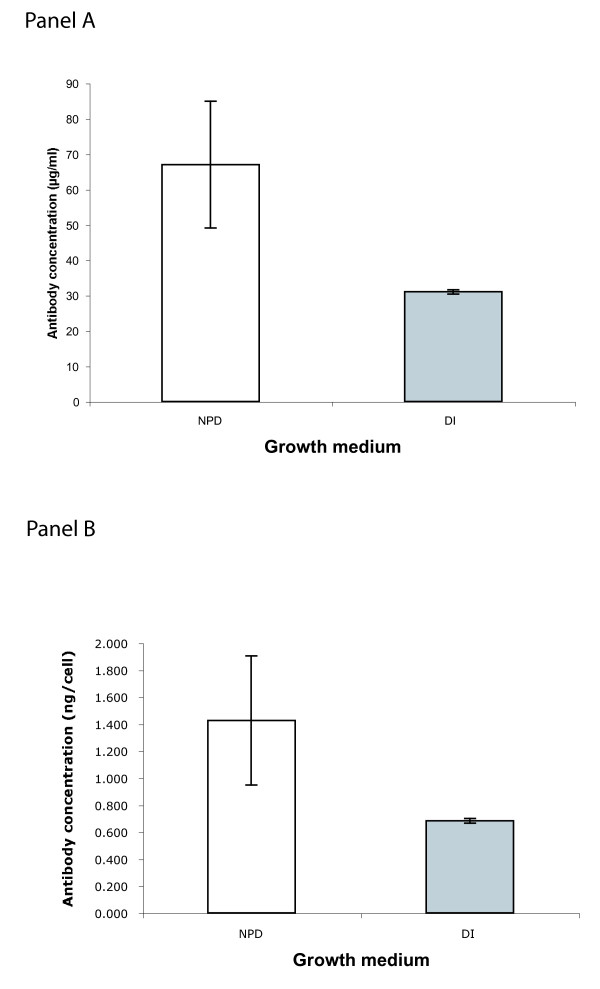
**IgM production by a stable hybridoma clone in 10% FCS**. Two parallel cultures were prepared in replicates from a stable hybridoma clone from our collection. One was grown in NPD and the other in DI medium and both were kept in standard culture conditions. After a week of growth the supernatants were collected, and the antibody concentrations were measured by a standard sandwich ELISA. Each column represents the mean antibody concentration that was measured in NPD and DI cultures. The error bars denote the standard error of the means. We have observed increased secretion of monoclonal antibody with a series of stable hybridoma clones and presented a detailed analysis with one of them in this manuscript. Panel A: Total antibody concentration measured in the culture supernatants; Panel B: Antibody concentration normalized per cell.

To study the effect of a NPD aqueous environment on secretion of monoclonal antibodies we studied the production of human monoclonal antibodies from several stabilized hybridoma clones. Hybridoma clones from our collection that have been stably producing antibodies for over 5 years were grown in DI based medium and then two parallel cultures were prepared from it, one in NPD and the other in DI based medium. Following a period of several days of adaptation, cells were seeded at equivalent densities in replicate and after five days of growth supernatants were harvested and antibody concentrations were measured by standard sandwich ELISA. The results of one of these experiments are presented in Figure 3 (panel A), although all showed similar results. As is evident from the graph, although the yields from the replicate NPD based cultures were somewhat variable (NPD culture concentrations ranged from 101 to 40 μg/mL, whereas in DI the range was much narrower: 30–32 μg/mL), there was overall a greater yield of monoclonal antibody in the NPD based media. However, some cells grow faster in NPD based media (see below). Thus this result might not reflect greater secretion per cell but rather greater proliferation of cells with similar secretion. To obviate this bias we normalized the antibody concentration to the number of cells in each culture (Figure 3, panel B). Following normalization the results are similar to the batch concentrations, and indicate that the secretion of monoclonal antibody in NPD based media is roughly twice that obtained in DI based media.

**Figure 4:**
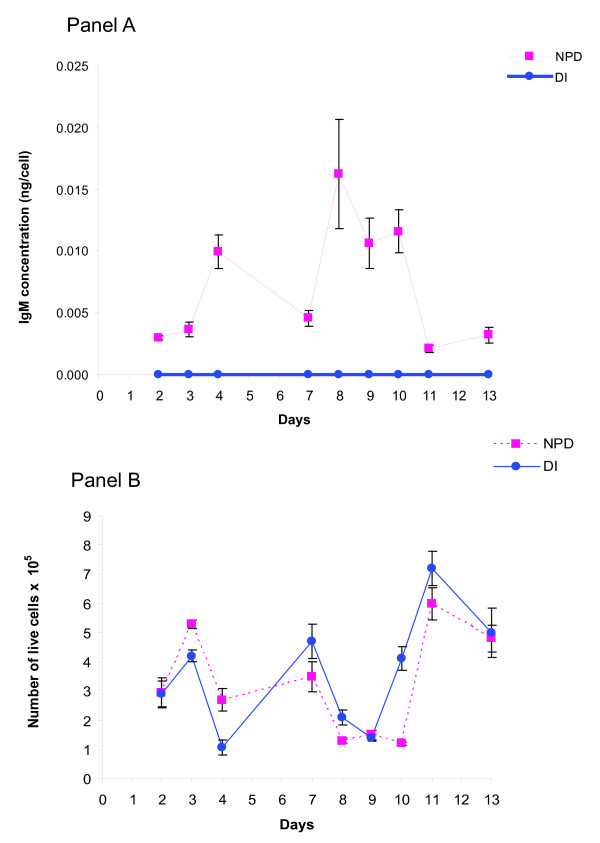
**IgM production by a stable hybridoma clone grown in 3% FCS**. Two cultures derived from the same culture of a stable hybridoma clone were grown, one in NPD and the other in DI based medium supplemented with 3% FCS. Before seeding the cells were washed in serum-free media to verify the removal of any residual serum. During a period of two weeks the supernatants were collected as indicated and the cells were counted on the same day. The cultures were fed on the 4^th ^and 10^th ^day and medium was placed in the cultures on day 6. Although the cells in DI culture proliferated normally under these conditions, they failed to produce measurable quantities of antibody. Panel A: IgM production per hybridoma cell in 3% FCS; Panel B: Number of live cells at each antibody titration.

To further study the effect of NPD media on secretion, we examined the secretion of monoclonal antibody in cultures grown in reduced serum. This experiment enabled us to examine secretion in cultures that were replicatively less active (relatively quiescent as compared to complete medium with 10% fetal bovine serum) but still metabolically active, thereby eliminating some of the proliferative bias of the NPD based media. Figure 4 presents the results of these experiments, where both daily antibody concentrations and viable cell counts of a stable hybridoma clone grown in 3% FCS, in replicate, were quantitated. In NPD culture the antibody concentration changed along with the quantity of viable cells in culture. Cell proliferation and variation in number was also a function of the replacement of medium and feedings (days 4 and 10 medium was added to the culture to feed cells and on day 6 the medium was completely replaced), which also impacted the concentration of antibody. In contrast, cells in the DI culture kept proliferating but failed to produce any measurable quantity of antibody. The graphs in Figure 4 depict typical relationships between hybridoma cell proliferation and the antibody content of the culture. In general, in NPD based media the quantity of antibody increases following an increase in cell number, which occurs following a proliferative burst after feeding. The pattern of the graphs reflects the dilutional effect on antibody concentration from media replacement and also the concomitant leap in cell proliferation (day 6 after medium replacement).

### Cell proliferation in a NPD based aqueous environment

The results of the previous experiments with the hybridoma clones suggested that NPD based media affected clonal expansion and survivability of human hybridoma cells. To further examine this hypothesis, we studied the growth of the immortal CHO cell line and primary human fibroblasts in NPD and DI based media.

#### Immortal cell lines grow faster in NPD water

**Figure 5:**
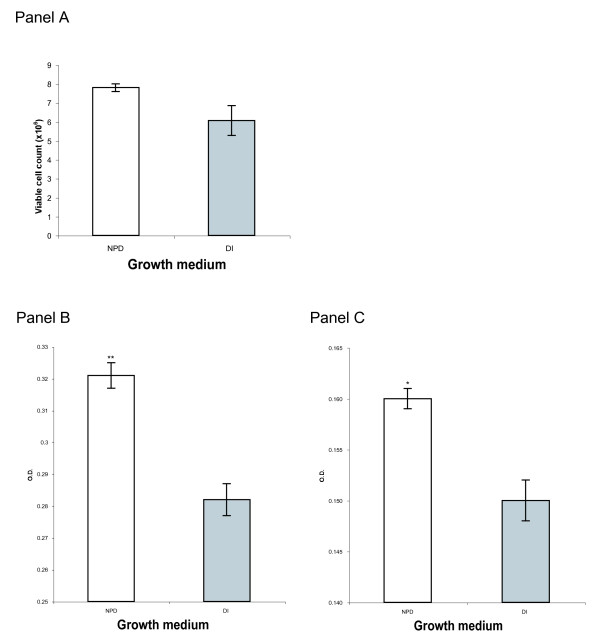
**Effect of serum reduction on CHO cell growth in NPD medium**. Panel A: CHO cell growth in complete medium: Cells were seeded at an initial density of 1.5 × 10^6 ^per 10-cm Petri dish in NPD and DI based medium in triplicates. After overnight growth they were detached by trypsinization and counted. The results are given as the number of viable cells. Each column represents a mean number of cells in each treatment. The error bars denote the standard error of the means. The difference between the treatments is 30%. The graph provides a representative result of an experiment, which was conducted with replicates and repeated three times. Panel B & C: CHO cell growth in reduced-serum medium: Cells were seeded in 96-well plates in multiple replicates (18 wells per treatment) in NPD or DI medium supplemented with 5% (Panel B) or 1% (Panel C) FCS. The results were quantified and analyzed by means of crystal violet dye retention assay. Each column represents the mean cell density following a given treatment in O.D. units. The error bars denote the standard error of the mean. *Significant difference between NPD and DI grown cells p = 0.0006, total difference 7%. **Significant difference between NPD and DI grown cells p = 0.0001, total difference 14%.

CHO cells were grown in NPD and DI based complete DMEM parallel cultures. Cells were seeded at an initial density of 1.5 × 10^6 ^per 10-cm Petri dish in replicate cultures. After overnight growth they were detached by trypsinization and counted. The results are presented in Figure 5, which demonstrates that in NPD medium the cells grew faster by an average of nearly 30%. To examine the effect of serum depletion on CHO cell growth, cells were seeded in parallel NPD and DI based cultures in replicate with either 5% or 1% FCS. In these experiments cell mass was quantitated by means of crystal violet dye retention assay [21]. The results of this experiment, illustrated in Figure 5 indicate that under serum reduced conditions cells grow faster in NPD based media as compared to a DI based media.

#### Primary human fibroblasts grow slower in NPD water

**Figure 6:**
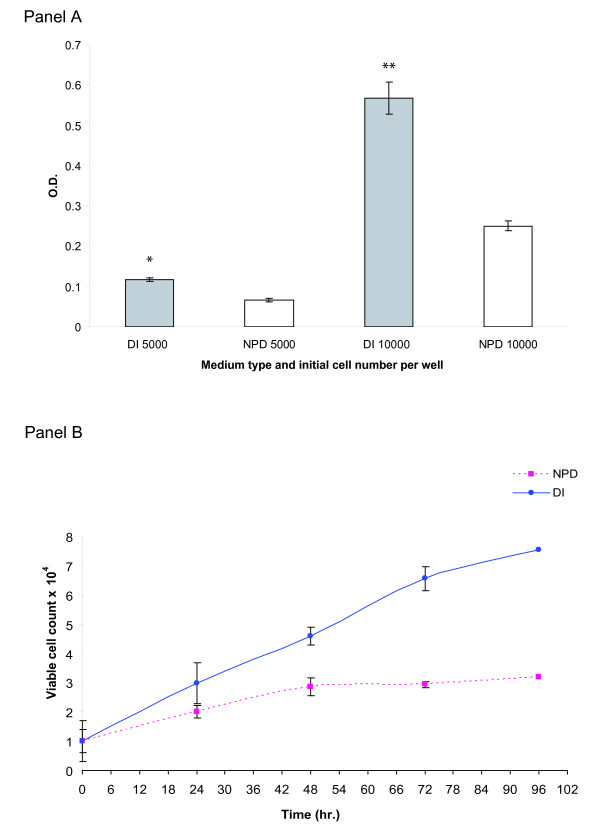
**Primary human fibroblast culture in NPD medium**. Panel A: Primary human fibroblast proliferation according to initial cell density: Primary human fibroblasts were seeded in replicate in a 96-well plate at two initial cell densities: five and ten thousand cells per well. After an overnight growth the cells were fixed and assayed by means of crystal violet dye retention method. The results are presented in O.D. values. Each column represents a mean O.D. of a given growth condition; the error bars denote the standard error of the mean. *Significant difference between DI and NPD for cell density of 5000 cells/well (p << 0.0001). **Significant difference between DI and NPD for cell density of 10000 cells/well (p << 0.0001). Panel B: Primary human fibroblast growth in complete medium: In a 24-well plate primary human fibroblasts were seeded in triplicate in NPD and DI based media. Next sets of triplicates (both in NPD and DI) were analyzed, by detaching and counting the viable cells, every 24 hours. The results are given in number of viable cells per well, the error bars denote the standard error of the mean.

Primary human fibroblasts at a relatively low passage (twenty population doublings) were first cultured in DI and NPD based media to adapt the cells to their respective growth media. Since primary fibroblasts are sensitive to cell density, we assessed the effect of NPD versus DI based media on cell proliferation with different initial seeding density. In a 96-well plate two cell densities were seeded in replicate wells in both NPD and DI based media, five and ten thousand cells per well. After an overnight growth the plates were analyzed with a crystal violet dye retention assay. The results of this assay are depicted in Figure 6, panel A. At both cell densities, fibroblasts grown in DI based media proliferated faster than in NPD based media. This difference was found to be highly statistically significant (p << 0.0001) The calculation of the percentage of a difference showed that at the higher density the difference between treatments was more pronounced (56%) than at the lower density (44%).

To further study the effect of NPD based media on primary human fibroblast growth, we studied the growth of fibroblasts in DI and NPD based media over eight days in replicate cultures. Cells were seeded at ten thousand cells per well in replicate parallel cultures, since in the previous experiment fibroblasts proliferated well at this density in DI based media. Growth curves from this experiment are displayed in Figure 6, panel B. As is evident from the curves, primary human fibroblasts proliferated poorly in NPD based media as compared to DI based media. This indicates that the environment in NPD water is less favorable for primary fibroblast outgrowth, and suggests that cell-cell sensing is somehow impaired since fibroblast proliferation is a function of cell density.

## Discussion

The process of isolating human monoclonal antibodies by the hybridoma technique is laborious and we are constantly seeking ways to optimize this process for increased yield of viable antibody secreting hybridoma clones, as well as for increased secretion of antibodies from the nascent clones for screening. To increase the efficiency of hybridoma isolation and monoclonal antibody production we tested NPD water as an alternative aqueous environment for cell culture. It demonstrated a positive effect on the hybridoma yield, which was statistically significant, and the fusion efficiency (portrayed by the number of viable hybridoma cells in each experiment) rose several fold in some cases. The variability of the enhancement (10–230%) was expected, as the fusion efficiency for human hybridoma formation typically differs between experiments, and depends on many factors that are largely host specific [1].

For production and therapeutic use of human monoclonal antibodies, efficient isolation of hybridoma clones and large-scale production and purification is required. We therefore tested the effect of NPD based media on outgrowth of hybridoma clones during the process of subcloning the primary hybridoma population and secretion of human monoclonal antibodies from hybridoma cells. Our results demonstrate a marked enhancement of NPD based media on efficiency of hybridoma clonal outgrowth and significantly increased antibody yield in NPD culture. The basis of these effects is likely multifactorial, however a direct effect of NPD based media on hybridoma clonability and some part of antibody synthesis and/or secretion is likely. Overall, there is an enhancement of antibody yield from a hybridoma culture regardless of whether it's caused by a higher per cell secretion rate or by a higher number of hybridoma cells.

To study the effect of NPD based media on cellular production and secretion alone, we decided to see if the picture would change if we slowed proliferation of the NPD and DI cultures through reduction of serum in the culture. The differences between antibody concentrations measured in cultures grown in 3% FCS were very dramatic. While the production of antibodies dropped in cultures grown in a NPD environment, the DI culture did not produce any measurable concentrations of antibody whatsoever. Interestingly, the number of cells in the cultures at various times post inoculation was similar. Therefore, it appears that NPD based media directly promotes production and secretion of antibody. We are currently studying this in more detail to determine if increased yields of antibody might result from a direct impact of NPD based media on secretion alone.

The propagation and growth of hybridoma cells under conditions of reduced serum diminishes antibody secretion by hybridoma cells [25-27]. However, secretion of human monoclonal antibodies in NPD based culture, under reduced serum conditions, suggests that production of these antibodies might be achieved in a more economical way. Both serum and serum free media formulations that contain growth factors are expensive and also require extensive purification. Use of NPD based media products, therefore, might facilitate purification and lower total cost of large-scale production.

Following the observation of a faster proliferation rate of hybridoma cells in a NPD environment, testing of standard cells in laboratory use was performed. As a model of an immortalized cell line, CHO cells were studied as they are widely used for production of protein products in both academic and commercial settings. Similar to hybridoma cells the CHO cells proliferate better in NPD based media as compared to DI media. In itself, this can be useful to enhance production of biopharmaceuticals, however, testing is under way to determine if there is a similar effect of NPD based media on secretion in CHO cell lines.

Unlike immortal cells, studies of primary human fibroblast growth in NPD based media revealed dramatically different results. Primary fibroblasts proliferated reproducibly slower in NPD based media. This effect on the rate of population doubling of the cells appears to have no impact on cell viability or the proliferative capacity of the primary fibroblasts to senescence (data not shown). Indeed, fibroblasts grown in NPD based media senesced at the same number of population doublings as fibroblasts from the same starter culture grown in DI based media. Since the cells in NPD media grew slower, they survived longer in culture chronologically.

The reverse effect of NPD based media on immortal cells and primary human fibroblasts is intriguing. Experiments performed herein demonstrate that primary human fibroblasts in NPD media "sense" a lower cell density as compared to DI based media. On the other hand, our fusion partner cell line, MFP-2, is of myeloma origin [1] and likely secretes autocrine factors that promote its own colony formation from a single cell. The local concentration of these substances is typically quite low in standard culture media, necessitating addition of exogenous growth factors from macrophage conditioned media (HCF) to promote clonal expansion. To explain these phenomena, we propose that the order imposed in a NPD based environment [12] establishes microenvironments in the "bulk" environment that disrupt cross-talk or inter-cellular communication. These microenvironments might effectively shield cells leading to a higher localized concentration of autocrine growth factors. This may explain the growth promoting properties of NPD based media on immortal cells, and at the same time the growth inhibitory effect on primary human fibroblasts.

Overall, our investigation of the impact of the aqueous environment on various aspects of cell biology suggests that *in vitro *biology might yield different results in a structured or altered aqueous environment, as opposed to the standard reverse osmosis water used in most laboratories. It supports the notion that environment should be considered in biological experimentation and applications [9,28]. Our studies support the working hypothesis that NPD water can play a significant role in cell biology and dramatically enhance the efficiency of processes that are of importance to bioprocessing and the biopharmaceutical industries. All together, our experiments suggest that a more detailed investigation of water structure, and its impact in all scientific disciplines, is warranted.

## Conclusion

We have demonstrated that NPD water can enhance hybridoma formation, cell proliferation, clonability and secretion. In addition, NPD based medium can enhance the viability of cultures under serum-reduced conditions. Finally, primary human fibroblasts proliferate poorly in NPD based medium and appear to sense a lower effective cell density in this environment. Taken together with the enhanced proliferation of hybridoma cells that likely secrete autocrine factors, these results support the hypothesis that NPD water is effectively composed of stabilized microenvironments.

## Authors' contributions

NG-Y coordinated the work of this project, performed much of the cell culture experiments and helped write the manuscript. EH & MT performed experiments relating to hybridoma production, subcloning and antibody secretion studies. VY, OK & LL designed all of these studies, participated in analysis of all data and participated in writing this manuscript. All authors read and approved the final manuscript.
